# Three‐dimensional mapping discovered arrhythmic substrate missed in the initial diagnosis of idiopathic ventricular fibrillation

**DOI:** 10.1002/ccr3.3918

**Published:** 2021-02-16

**Authors:** Yoshihiro Harano, Keita Masuda, Shunsuke Inoue, Yuto Iioka, Shinya Kowase, Jun Osada, Kazuhiko Yumoto

**Affiliations:** ^1^ Department of Cardiology Yokohama Rosai Hospital Yokohama Japan

**Keywords:** catheter ablation, idiopathic ventricular fibrillation, mapping, substrate, ventricular tachycardia

## Abstract

During an initial diagnosis of IVF, an arrhythmic substrate may be missed for several reasons such as lack of information; thus, a careful follow‐up is important. A three‐dimensional mapping may identify a possible missed arrhythmic substrate in IVF.

## Graphical abstract

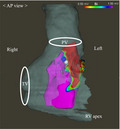

## 1 CASE REPORT

Management of idiopathic ventricular fibrillation (IVF) is challenging because an arrhythmic substrate may be missed at initial diagnosis; therefore, careful follow‐up and reassessment are necessary. Three‐dimensional mapping would be useful for reassessment of IVF because it revealed an arrhythmic substrate in a patient, 12 years after his initial IVF diagnosis.

**FIGURE 1:**
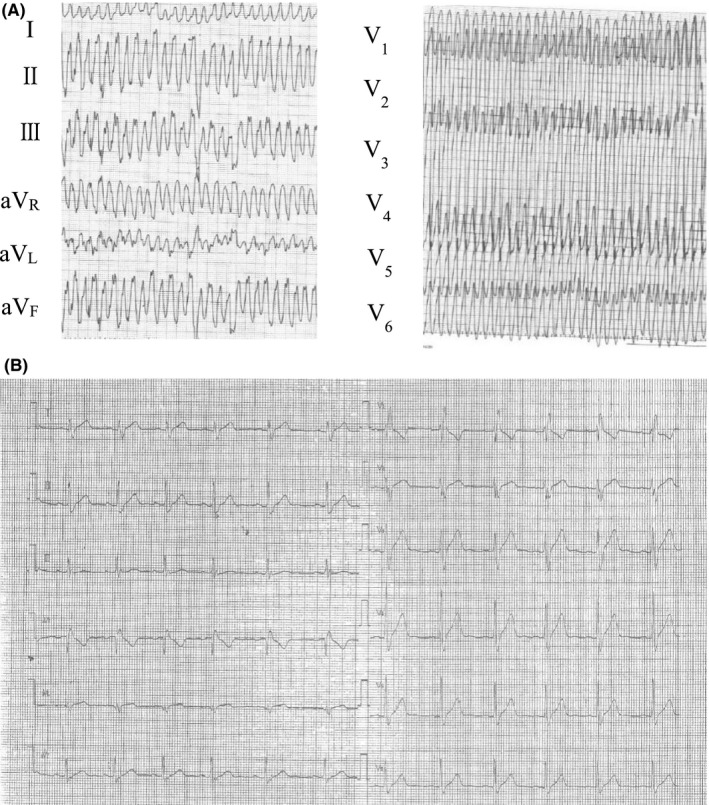
A, Electrocardiogram on the patient's first admission when he was 33 y old. Fast ventricular tachycardia including a partially polymorphic waveform and a heart rate of 330 beats per min were recorded. B, Electrocardiogram at rest. Right bundle branch block was observed

Twelve years ago, a 33‐year‐old man (now 45 years old) with no significant family history and a medical history of syncope when playing golf developed sudden cardiac arrest (SCA) after drinking alcohol. His heartbeat was restored by an automated external defibrillator, and a fast ventricular tachycardia (VT) with a short cycle length (CL), including a partially polymorphic waveform, was recorded at another hospital (Figure 1A). Thus, cardiologists at the previous hospital judged that this patient had VT or ventricular fibrillation (VF) at that time. Although various examinations including cardiac magnetic resonance imaging (MRI), coronary angiography, echocardiogram, and a pilsicainide provocation test were performed, the underlying heart disease was not clear. Also, the electrocardiogram (ECG) at rest showed no specific findings except a complete right bundle branch block (Figure 1B). The patient was diagnosed with idiopathic ventricular fibrillation (IVF) by the cardiologists at the previous hospital. An implantable cardioverter defibrillator (ICD) was not implanted because of his rejection. Amiodarone was also recommended, but he refused the prescription because of the possible side effects. Thus, he began oral medication with bisoprolol 2.5 mg per day for the arrhythmia. However, 5 years later, a second SCA occurred due to the same ventricular arrhythmia, and he received an ICD (Dual‐chamber ICD, mode DDI) implantation after successful resuscitation.

**FIGURE 2:**
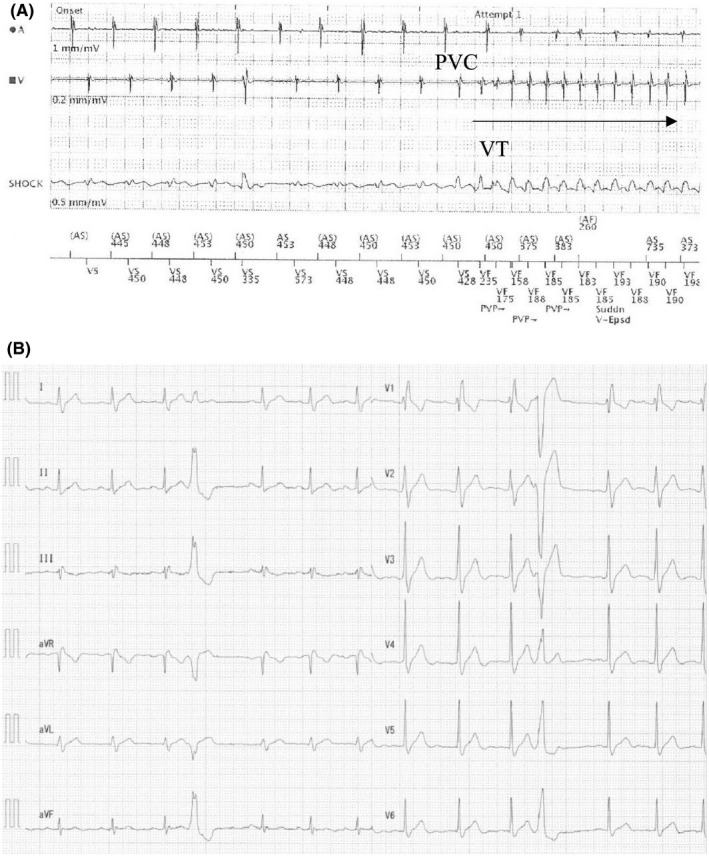
A, Intracardiac implantable cardioverter defibrillator record of a monomorphic ventricular tachycardia (VT). A monomorphic VT (mean cycle length, 185 ms) was recorded and consistently triggered by the same premature ventricular contraction (PVC). B, Electrocardiogram at rest before the first ablation therapy. Complete right bundle branch block and a single PVC of a left bundle branch block configuration with an inferior axis observed

**FIGURE 3:**
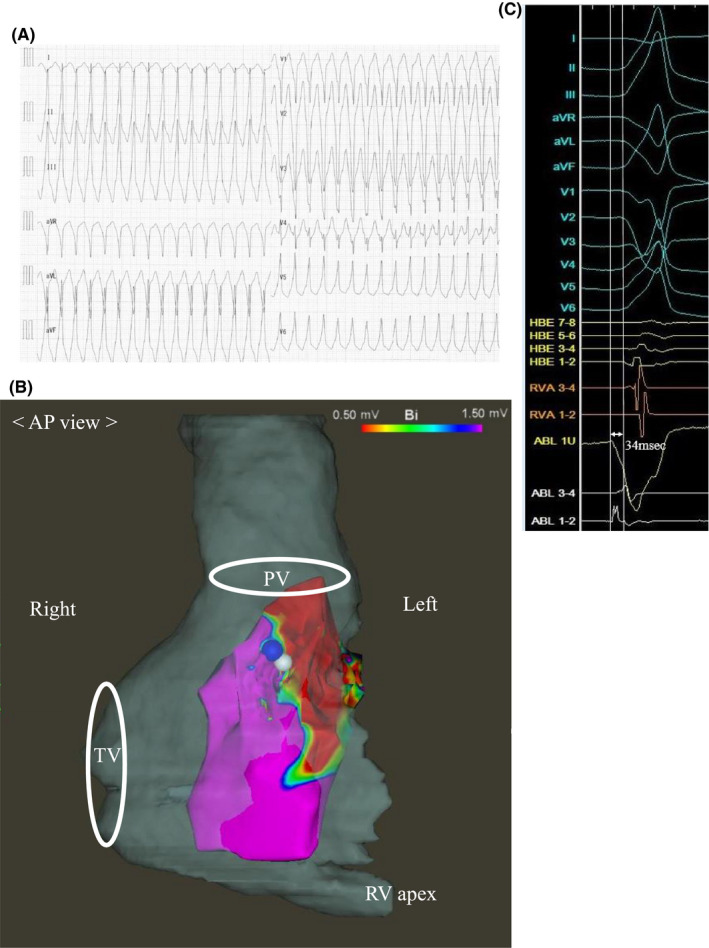
A, Electrocardiogram on admission during a ventricular tachycardia (VT) storm before the third ablation therapy. The morphology of the VT was a left bundle branch block configuration with an inferior axis. Its estimated origin was the right ventricular outflow tract. B, Three‐dimensional (3D) voltage map during sinus rhythm. Anterior to posterior view of the 3D mapping of the right ventricle in the third ablation session is superimposed on a preoperative figure of Computed Tomography. Blue tag indicates a successful ablation site for premature ventricular contraction and ventricular tachycardia. White tag indicates a good pacemap with a score of 96. AP view, anterior‐posterior view; PV, pulmonary valve; TV, tricuspid valve; RV apex, right ventricular apex. C, Intracardiac electrogram of the success site during the third ablation therapy. The distal part of the ablation catheter (ablation [ABL] 1 U and 1‐2) detected depolarization 34 ms earlier than any other leads of the electrocardiogram. HBE, His bundle electrogram; RVA, right ventricular apex

Twelve years later, the patient was admitted to our hospital for frequent ICD shocks. His ICD record showed rapid monomorphic VT (mean CL, 185 ms), which was always triggered by the same premature ventricular contraction (PVC) (Figure 2A). The morphology of the PVC was a left bundle branch block configuration with an inferior axis; catheter ablation of the trigger PVC was performed (Figure 2B). A three‐dimensional (3D) voltage map of the right ventricle was constructed, and a low‐voltage area (LVA) on the free wall of the right ventricular outflow tract (RVOT) was detected. However, only a few PVCs were observed during the catheter ablation, and we performed a pacemap‐guided ablation of the PVC. A good pacemap was obtained at the border zone of the LVA, and the target PVC was ablated at the site. Shortly after discharge, he experienced a recurrence of ICD shocks and underwent a second ablation session the following month. However, the second ablation failed again because very few PVCs were observed, and he revisited our hospital a month after the second session for frequent ICD shocks. Monomorphic VT originating from the RVOT was recorded on an ECG (Figure 3A), and a third ablation session for recurrent VT was performed. The morphology of the VT was the same as the trigger PVC, which was frequently observed during his sessions. An almost perfect pacemap (score, 96) with PVC was obtained near the LVA, slightly posterior to the prior ablation site. The ventricular potential of the PVC on the ablation catheter showed up earlier than the QRS onset in any leads on the ECG at this site (Figure 3B‐C). Both VT and PVC were successfully eliminated and not inducible after ablation. There were no ICD shocks one‐and‐a‐half years after the last session.

## 2 DISCUSSION

In this case, the recorded ventricular tachyarrhythmia on the ICD log at our hospital was monomorphic VT, not VF. On the other hand, the recorded log from the other hospital after SCA was fast VT including a partially polymorphic waveform. Thus, the cardiologists in that hospital judged that the cause of his SCA was VT or VF and diagnosed him with IVF. This difference could be explained as follows. VF has been reported to be sometimes promoted by VT accompanied with hemodynamic instability and can develop into VF or SCA.^1^ Most VTs originating from the RVOT are considered as benign arrhythmias; however, some VTs can be malignant and develop into VF or polymorphic VT; the characteristics of these malignant VTs are more likely to occur in patients with a history of syncope and short CL.^2^ This patient had several events of syncope of unknown cause and monomorphic VT with a very short CL (185 ms). Therefore, we estimated that his repetitive SCAs were primarily due to a malignant type of VT originating from the RVOT.

When a patient with SCA is taken to a hospital, there could be an initial lack of clinical history and information; therefore, the initial diagnosis of IVF might be incomplete. In fact, it has been reported that more than one‐fifth of patients initially diagnosed with IVF are re‐diagnosed with another specific disease; hence, continuous follow‐up and reassessment of patients with IVF has become very important.^3^ This patient presented to our hospital with IVF and was uneventfully followed‐up in the ICD clinic for several years. When multiple ICD shock events due to monomorphic VT were detected, we suspected that his diagnosis was possibly not IVF. In the subsequent ablation session, 3D mapping revealed the existence of an LVA on the RVOT, which showed that he had a structural arrhythmic substrate. Moreover, the induced ventricular arrhythmia, which was reproducibly a monomorphic VT, showed a regular fixed sequence of ventricular potentials that arose from the LVA. Ablation of the LVA resulted in the successful elimination of the target PVC and VT. Based on these results, the patient was finally diagnosed with a scar‐related VT, not IVF.

Although cardiac biopsy and genetic examination were not performed, we ruled out other specific heart diseases before ablation therapy. For example, his echocardiogram, cardiac MRI, and pilsicainide infusion test did not show any positive features of Brugada syndrome. Further, the present case did not match the current diagnostic criteria of arrhythmogenic right ventricular cardiomyopathy.^4^ For analyzing other underlying diseases, cardiac biopsy, and DNA testing could have been performed; however, as cardiac biopsy for unexplained ventricular arrhythmia is not strongly recommended,^5^ we did not perform it. Moreover, a DNA test was not performed because he was less likely to have genetic arrhythmia based on his 12‐lead ECG at rest and family history.

Our patient who was initially diagnosed with IVF was eventually re‐diagnosed with a scar‐related VT via 3D mapping. In most cases, 3D mapping has been rarely performed as one of the initial diagnostic tests for IVF.^6^ Therefore, we suggest that a 3D mapping system might be useful in finding a missed substrate and in clarifying another specific arrhythmic disease like in the present case.

## 3 CONCLUSION

We diagnosed a scar‐related VT based on 3D mapping results in a patient who was initially diagnosed with IVF 12 years ago. When managing patients with IVF, continuous careful follow‐up is very important, and 3D mapping could be useful for the reassessment of IVF because an arrhythmic substrate might have been missed.

## CONFLICT OF INTEREST

The authors state that they have no financial conflict of interest.

## AUTHOR CONTRIBUTIONS

KM: elaborated this case report and drafted the manuscript. SI and YI, and JO: attended ablation session together of the present case and contributed their clinical success. SK and KY: conceived of this case report and gave me advice to improve this manuscript. All authors read and approved the final manuscript.

## ETHICAL APPROVAL

All the authors collectively agree that the manuscript is original and has not been published elsewhere.

## Data Availability

The data is available from the corresponding author for reasonable request.
